# Surgical treatment of chronic Achilles tendon rupture results in improved gait biomechanics

**DOI:** 10.1186/s13018-022-02948-2

**Published:** 2022-02-02

**Authors:** Anna Nordenholm, Eric Hamrin Senorski, Olof Westin, Katarina Nilsson Helander, Michael Möller, Jón Karlsson, Roland Zügner

**Affiliations:** 1 Department of Health and Rehabilitation, Institute of Neuroscience and Physiology, Sahlgrenska Academy, University of Gothenburg, Medicinaregatan 11-13, 41390 Gothenburg, Sweden; 2 Department of Orthopaedics, Institute of Clinical Sciences, Sahlgrenska Academy, University of Gothenburg, Gothenburg, Sweden

**Keywords:** Gait analysis, Three-dimensional, Late detected, Missed diagnosis, Delayed treatment

## Abstract

**Background:**

Chronic Achilles tendon rupture is associated with persistent weakness at push-off with the affected foot and poor balance, resulting in significant alterations to normal gait. Surgical repair is the most common treatment for improving gait in patients with a Chronic Achilles tendon rupture, but, to date, the outcomes have not been quantified in the literature.

**Methods:**

A total of 23 patients with a Chronic Achilles tendon rupture (mean age 61 ± 15 years) underwent three-dimensional gait analysis according to a standardized protocol using an optical tracking system. Data of spatiotemporal, kinematic and kinetic variables were collected preoperatively and one year postoperatively. In addition, the postoperative gait biomechanics were compared with the gait biomechanics of a control group consisting of 70 healthy individuals (mean age 49 ± 20 years). The prospectively collected data were analyzed by an independent *t* test.

**Results:**

Postoperatively, increments were found in gait speed (mean difference − 0.12 m/s), stride length (− 0.12 m), peak ankle moment (− 0.64 Nm/kg), peak ankle power (− 1.38 W/kg), peak knee power (− 0.36 m) and reduced step width (0.01 m), compared with preoperative gait biomechanics (*p* < 0.014). Compared with the control group, patients with a Chronic Achilles tendon rupture exhibited slower postoperative gait speed (mean difference 0.24 m/s), wider step width (− 0.02 m), shorter stride length (0.16 m), longer relative stance phase (− 2.15%), lower peak knee flexion (17.03 degrees), greater peak knee extension (2.58 degrees), lower peak ankle moment (0.35 Nm/kg), peak ankle power (1.22 W/kg) and peak knee power (1.62 W/kg), (*p* < 0.010).

**Conclusion:**

Surgical intervention and postoperative rehabilitation can be an effective treatment for alterations in gait after a Chronic rupture of the Achilles tendon. However, at one year postoperatively, patients still exhibit impairments in spatiotemporal variables and knee and ankle power compared with healthy controls.

## Introduction

The incidence of Achilles tendon ruptures (ATR) peaks in individuals between 30 and 50 years, but a second peak phenomenon has also been found after the age of 60 years [1, 2]. An ATR is primarily diagnosed on the basis of patient history and physical examination and commonly occurs momentarily during high-intensity sporting activities, but can also occur gradually with less intense injury mechanisms [2–4]. Up to 25% of ATRs are missed, either due to a misdiagnosis by the physician or as a result of the patient misinterpreting the injury and not seeking immediate care [5, 6]. In cases with an atypical patient history and an absence of a major trauma, the assessment is more difficult and may result in a misdiagnosis, typically an ankle sprain or calf muscle tear [5–8].

An ATR that is left untreated results in persistent weakness at push-off with the affected foot/ankle and is defined as “chronic” (CATR) when the diagnosis and adequate treatment are delayed for 4 weeks [5, 9, 10]. Non-surgical treatment may contribute to improvements, but the current consensus is that surgical repair is the preferred treatment for improving muscular function and walking ability [5, 10–12]. The repair of a CATR is more demanding than an early suture repair due to the retraction of the ruptured tendon ends and the degenerated status of the tissues [5, 10, 11].

Several studies have previously reported on the long-term effect on gait biomechanics after an acute ATR, independent of surgical or non-surgical treatment [13–16]. Remaining side-to-side differences are commonly reported in patients with ATR in terms of the peak plantar flexion moment and peak plantar flexion power [14, 15]. When compared with healthy controls, increased dorsal flexion and decreased plantar flexion range of motion (ROM), as well as impaired ankle muscle peak power on the injured side, have been reported at 2–5 years after an acute ATR [15]. Similar bilateral effect on gait biomechanics is most likely found also in patients with CATR; however, this has not yet been studied.

The current literature suggests that gait biomechanics gradually improves after treatment for an acute ATR, but it does not necessarily normalize [13–17]. The clinical experience of orthopedic surgeons and physical therapists is that patients with a CATR benefit from surgical intervention. However, to date, these improvements have not been quantified in the literature. The aims of this study were to investigate whether the gait biomechanics in patients with a CATR can be improved after surgical intervention and to compare these patients’ gait at one year postoperatively with those of healthy controls. The hypothesis was that surgical intervention would result in improvements in gait biomechanics at one year postoperatively compared with the preoperative status.

## Material and methods

### Patients

The study was approved by the regional ethical review board in Gothenburg (reference number 554–15, 2015–09-30). All the patients received oral and written information about the study and were informed of their right to withdraw from the study at any time without explanation. The patients signed a written consent to participate in the study. The healthy control group gave oral consent for their data to be used at group level. Patients referred from the health care center to units with specialized orthopedic surgeons (MM, KNH) at Sahlgrenska University Hospital and Kungsbacka Hospital in 2014–2016 and were scheduled for surgical intervention for a CATR were randomly asked to participate in the study. The inclusion criteria were patients with a unilateral CATR defined as an ATR that had been left untreated for at least four weeks. The first 24 patients who agreed to participate were included in the study and participated in both pre- and postoperative gait analysis. During the statistical analysis, the authors found that one patient had been incorrectly included in the study since too short a time had passed since the ATR, after which that patient was excluded from the statistical analysis and a total of 23 patients with a CATR were included in the study. In addition, 70 healthy individuals who are included in the gait laboratory’s database served as a control group and all confirmed that they had no effect on walking ability. The demographics of the CATR group are found in Table 1.

**Table 1:** Patient demographics

	CATR group (*n* = 23)	Healthy controls (*n* = 70)	Mean difference (95% CI)	*P* value
Patient sex				
Male (%)	15 (65)	36 (51)		0.29
Female (%)	8 (35)	34 (49)		
Age				
Mean (SD)	61 (15)	46 (20)	15 (6.3 to 24.3)	< 0.001
Median (range)	66 (28–83)	47 (13–84)		
Weight (kg)				
Mean (SD)	83 (15)	75 (13)	9 (2 to 15)	0.017
Median (range)	84 (55–116)	72 (54–115)		
Height (m)				
Mean (SD)	1.73 (0.09)	1.74 (0.09)	0.01 (− 0.05 to 0.04)	0.65
Median (range)	1.75 (1.51–1.89)	1.75 (1.57–1.92)		
BMI				
Mean (SD)	28 (4.78)	25 (4.54)	3.1 (1.2 to 4.9)	0.008
Median (range)	27 (21–40)	24 (18–35)		

*BMI* body mass index, *CATR* Chronic Achilles Tendon Rupture, *CI* confidence interval, *kg* kilograms, *SD* standard deviation

### Surgical treatment

The patients received surgical treatment between one and 36 months (mean 9, median 7 months) after the ATR from one of two experienced orthopedic surgeons. The ability to make the correct diagnosis in this patient group is very different, which can lead to a wide delay in the referral of a patient to an orthopedic specialist. Twenty-two of the 23 patients with a CATR were treated with augmentation using a free flap from the gastrocnemius aponeurosis, a surgical technique previously described by Nilsson Helander et al. [18]. One of the patients also received a suture anchor in the calcaneus due to the distal location of the rupture. In one patient, a free semitendinosus autograft was used instead, due to the large size of the gap. Postoperatively, a below-knee plaster cast was used for three to five weeks, followed by an adjustable lower-leg brace (DonJoy ROM Walker), which was removed eight weeks after surgery. Partial weight-bearing was allowed after three weeks and was gradually increased to full weight-bearing in the brace, which was allowed after six weeks. The patients were scheduled for and referred to physical therapy for more specific criteria-based exercise therapy and load instructions at eight weeks postoperatively.

### Gait analysis

The gait analysis was performed preoperatively (mean 2 months, range 1–9 months after injury) and one year postoperatively using an optical tracking system (OTS). All the gait analyses were performed according to a standardized protocol, where the test subject wore underwear and walked barefoot on the floor. A total of 15 spherical markers (ø 12 mm) were attached to the skin of the lower extremities and the pelvis with double-adhesive tape by an experienced examiner (RZ), according to a skin marker model based on Kit Vaughan and presented in detail by Weidow et al. [19] and validated by Tranberg et al. [20] and Zügner et al. [21, 22]. Markers were attached to the proximal border of the sacrum, anterior and superior iliac spine, lateral knee joint line, proximal boarder of the patella, tibial tubercle, tuber calcanei, lateral malleolus and between the second and third metatarsals. A modified Coda pelvis was used to define the pelvis segment [23]. The modification consisted of a reduction of the two bilateral markers on the posterior superior iliac spine that were replaced by one marker at the mid-point of the proximal border of the sacrum.

For data acquisition, a 16-camera motion capture system with a sampling rate of 240 Hz (Oqus 700+, Qualisys AB, Göteborg, Sweden), together with 4 force plates (Amti Optima OPT400600-HF-2K-CTT), was used. A static recording with the test subject standing in an upright position in the calibrated volume aligned to the global coordinate system was performed prior to the gait analysis in order to scale the subject’s anthropological measurements in relation to the marker positions. The test subjects were then asked to walk 5–10 times at a self-selected speed through the calibrated volume to familiarize themselves with the situation and then to perform six gait trials of which the approved trials for each test subject (median 5, range 1–6) were selected for further evaluation. The mean of approved trials for each test subject was used in the analysis to increase the reliability of the testing. In order not to miss valuable data, patients with few preoperative trials were also included. A trial was excluded from the analysis if the patient missed stepping on the force plates correctly or due to other technical problems. The spatiotemporal variables that were collected were speed (m/s), step width (m), stride length (m) and stance phase (% of total gait cycle relative to swing phase). The kinematic variables were degrees of dorsi- and plantar flexion together with flexion and extension in the knee joints during stance phase. Foot progression in the horizontal plane (degrees) was calculated using to the global laboratory coordinate system. Kinetic variables collected in the sagittal plane during the stance phase were power (W/kg) and moment (Nm/kg) in the ankle and knee joints. Prior to any calculations, the marker data obtained from the recordings were filtered using a Butterworth fourth-order filter with a cutoff frequency of 6 Hz. For calculations of spatiotemporal, kinematic and kinetic variables, Visual 3D™ software (C-Motion, Inc., Germatown, USA) was used.

### Statistical analysis

Gait variables of the CATR extremity were used for the subsequent analysis and, in the control group, data from the right side were used. The mean of the approved trials for each patient was used for analysis. Descriptive data were reported as the mean (standard deviation, SD) and median (range). All the variables were approximately normally distributed, as assessed by a visual inspection of the histograms. A paired *t* test was used to analyze the differences (within the same subject) between preoperative and postoperative gait biomechanics. For comparison between the patient sample and the control group, an independent *t* test was performed. Effect size was calculated using Cohen’s *d*, with the standard deviation of the difference as the standardizer and interpreted using the criteria of 0.2 = small, 0.5 = medium and 0.8 = large effect [24]. All the tests were two-tailed, and alpha was set at 0.05. Post hoc power calculations were performed on the pre- and postoperative differences for included variables (23 patients). The statistical power ranged between 4 and 99%, with four variables exceeding 80% power; stride length, peak ankle moment, peak ankle power and peak knee power.

## Results

### Pre- versus postoperative gait biomechanics

**Fig. 1:**
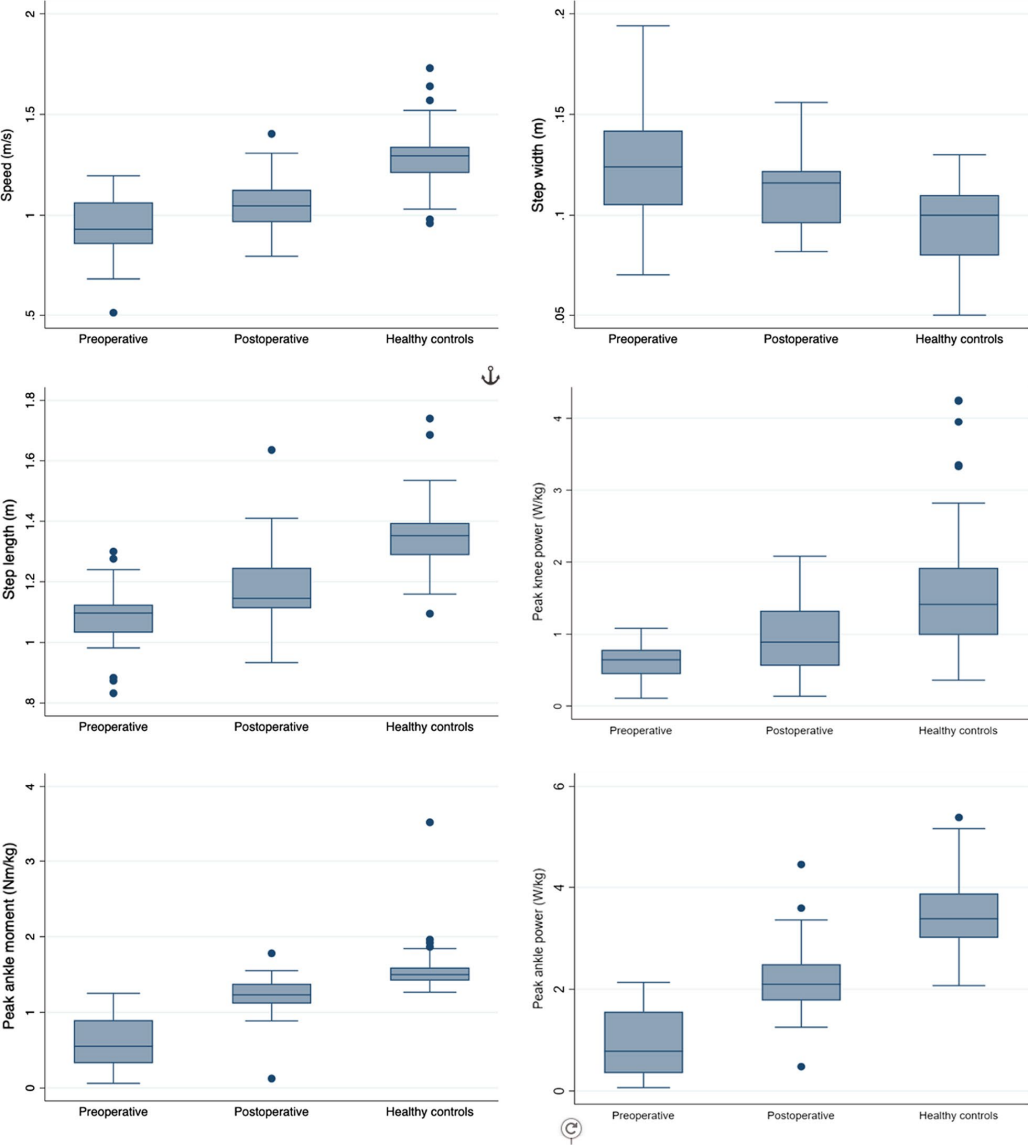
Boxplots of gait variables that were significantly improved in patients with chronic Achilles tendon rupture at one year after surgical intervention, including comparison with healthy controls

Several gait variables improved after surgical intervention. The patients with a CATR exhibited a significantly reduced step width, increased speed, stride length, ankle moment and ankle and knee power compared with the preoperative status (Fig. 1; Table 2). Spatial gait variables, knee kinematics and power development in the ankle joint were still impaired compared with healthy controls. Gait speed and stride length increased postoperatively, with a mean difference of − 0.12 m/s (*p* = 0.013) and − 0.12 m (*p* = 0.002), respectively. Step width decreased by a mean difference of 0.01 m (*p* = 0.014). The effect sizes for significant differences were medium (Cohen’s *d* 0.56–0.74). There were no differences in kinematics between the pre- and postoperative evaluations (Table 2). Peak ankle moment increased by a mean difference of − 0.64 Nm/kg (*p* < 0.001), peak ankle power by a mean difference of − 1.38 W/kg (*p* < 0.001) and peak knee power by a mean difference of − 0.36 W/kg (*p* = 0.003) (Table 2). The effect sizes for significant differences ranged from medium to large (Cohen’s *d* 0.70–1.38).

**Table 2:** Gait variables: pre- and postoperative and comparison with healthy controls

	Preop. CATR (*n* = 23)	Postop. CATR (*n* = 23)	Pre- versus postop. CATR			Healthy controls (*n* = 70)	CATR postop. versus healthy controls		
	Mean ± SD	Mean ± SD	Mean diff. ± SD (CI)	*P* value^a^	Effect size (CI)	Mean ± SD (CI)	Mean diff. (CI)	*P* value^b^	Effect size (CI)
Approved gait trials	4.7 ± 1.1 (1 to 6)	4.7 ± 0.8 (3 to 6)							
*Spatiotemporal variables*									
Speed (m/s)	0.93 ± 0.15	1.05 ± 0.14	− 0.12 ± 0.21^a^ (0.21 to 0.03)	**0.013**	0.56** (− 1.00 to − 0.12)	1.29 ± 0.14	0.24^b^ (0.17 to 0.30)	** < 0.001**	1.73*** (1.19 to 2.26)
Step width (m)	0.12 ± 0.03	0.11 ± 0.02	0.01 ± 0.02^a^ (0.00 to 0.02)	**0.014**	0.56** (0.11 to 0.99)	0.10 ± 0.02	− 0.02^b^ (− 0.03 to − 0.01)	**0.002**	0.79** (− 1.27 to − 0.30)
Step length (m)	1.08 ± 0.11	1.19 ± 0.14	− 0.12 ± 0.16^a^ (− 0.18 to − 0.05)	**0.002**	0.74** (− 1.20 to − 0.27)	1.36 ± 0.11	0.16^b^ (0.10 to 0.22)	** < 0.001**	1.36*** (0.85 to 1.87)
Stance phase (%)	63.52 ± 2.49	63.36 ± 1.74	0.16 ± 2.05^a^ (− 0.72 to 1.05)	0.704	0.08 (− 0.33 to 0.49)	61.21 ± 1.62	− 2.15^b^ (− 2.94 to − 1.36)	** < 0.001**	1.31*** (− 1.81 to − 0.80)
*Kinematics*									
Peak ankle dorsal flexion (degrees)	16.08 ± 3.54	13.9 ± 3.82	2.11 ± 5.09^a^ (− 0.08 to 4.31)	0.059	0.42 (− 0.02 to 0.84)	14.11 ± 5.44	0.15^b^ (− 2.29 to 2.57)	0.905	0.03 (− 0.42 to 0.50)
Peak foot progression (degrees)	− 16.09 ± 10.48	− 13.82 ± 7.73	− 2.27 ± 5.49^a^ (–4.65 to 0.10)	0.060	0.41 (− 0.84 to 0.02)	− 13.64 ± 6.63	0.17^b^ (− 3.13 to 3.47)	0.917	0.03 (− 0.45 to 0.50)
Peak knee flexion (degrees)	38.35 ± 8.98	40.25 ± 5.74	− 1.90 ± 8.28^a^ (− 5.48 to 1.68)	0.283	0.23 (− 0.64 to 0.19)	57.28 ± 4.41	17.03^b^ (14.75 to 19.31)	** < 0.001**	3.57*** (2.87 to 4.27)
Peak knee extension (degrees)	− 0.04 ± 4.42	− 0.66 ± 5.45	0.62 ± 3.41^a^ (0.86 to 2.10)	0.392	0.18 (− 0.23 to 0.59)	1.92 ± 3.56	2.58^b^ (0.62 to 4.53)	**0.010**	0.63* (0.15 to 1.11)
*Kinetics*									
Peak ankle moment (Nm/kg)	0.57 ± 0.35	1.21 ± 0.31	− 0.64 ± 0.47^a^ (− 0.84 to − 0.44)	** < 0.001**	1.38*** (− 1.94 to − 0.79)	1.56 ± 0.29	0.35^b^ (0.21 to 0.49)	** < 0.001**	1.18*** (0.68 to 1.68)
Peak ankle power (W/kg)	0.86 ± 0.61	2.24 ± 0.83	− 1.38 ± 1.08^a^ (− 1.85 to − 0.91)	** < 0.001**	1.28*** (− 1.82 to − 0.71)	3.46 ± 0.73	1.22^b^ (0.86 to 1.58)	** < 0.001**	1.61*** (1.09 to 2.14)
Peak knee moment (Nm/kg)	0.48 ± 0.16	0.50 ± 0.24	− 0.01 ± 0.21^a^ (− 0.10 to 0.08)	0.754	0.07 (− 0.47 to 0.34)	0.44 ± 0.12	− 0.04^b^ (− 0.13 to 0.02)	0.174	0.33 (− 0.80 to 0.15)
Peak knee power (W/kg)	0.63 ± 0.27	0.99 ± 0.54	− 0.36 ± 0.52^a^ (− 0.59 to − 0.14)	**0.003**	0.70** (− 1.15 to − 0.23)	1.61 ± 0.85	0.62^b^ (0.24 to 0.99)	**0.001**	0.79*** (0.30 to 1.27)

*CATR* Chronic Achilles Tendon Rupture, *CI* confidence interval, *kg* kilograms, *m* meter, *m/s* meters per second, *Nm* Newton meter, *Preop* preoperative, *Postop* postoperative, *SD* standard deviation, *W* Watt

*Small effect, **Medium effect, ***Large effect

^a^Paired *t* test ^b^Independent *t* test. Significant differences in bold text

### Comparison with healthy controls

There were significant differences between the CATR patients and the healthy controls in terms of age, weight and BMI (Table 1). All the spatiotemporal variables differed between patients with a CATR and healthy controls at the follow-up (Table 2). Patients with a CATR exhibited slower gait speed, with a mean difference of 0.24 m/s (*p* < 0.001), a wider step width, with a mean difference of − 0.02 m (*p* = 0.002), a shorter stride length, with a mean difference of 0.16 m (*p* < 0.001), and a longer relative stance phase, with a mean difference of − 2.15%, *p* < 0.001), compared with the healthy control group. The effect sizes for significant differences ranged from medium to large (Cohen’s *d* 0.79–1.73). Patients with a CATR exhibited a lower peak knee flexion angle, with a mean difference of 17.0 degrees (*p* < 0.001), and a greater peak knee extension, with a mean difference of 2.6 degrees *p* < 0.010), compared with healthy controls. Effect sizes for significant differences ranged from small to large (Cohen’s *d* 0.63–3.57). Patients with a CATR also exhibited lower peak ankle moment, with a mean difference of 0.35 Nm/kg (*p* < 0.001), peak ankle power, with a mean difference of 1.22 W/kg (*p* < 0.001), and peak knee power, with a mean difference of 1.62 W/kg (*p* = 0.001) compared with healthy controls (Table 2). The effect sizes for significant differences were large (Cohen’s *d* 0.79–1.61).

## Discussion

The most important finding of this study was that patients with a CATR exhibited improved gait biomechanics at one year following surgical intervention and postoperative rehabilitation. There were significant improvements in terms of gait speed, stride length, step width and peak power in the knee and ankle joints. Gait speed, step width, stride length, stance phase and knee and ankle power still differed significantly between compared with the healthy controls at the follow-up.

The purpose of surgical intervention for a CATR is to create the prerequisites for improved ankle function and gait, which, according to the current consensus, is difficult without surgery [10, 11]. This study shows that surgical intervention, followed by postoperative rehabilitation after a CATR, results in several improvements in gait biomechanics at one year postoperatively. The greatest improvements were found in terms of ankle flexor peak moment and power, which means, in clinical terms, an improved push-off ability during gait [25]. Increased knee and ankle joint peak power, which is the product of moment and angular velocity, increase the ability to absorb and generate power in the ankle and knee joints throughout the stance phase and generate greater maximum power in the terminal stance phase. Preoperatively, patients with a CATR exhibited a lower generated peak power in the ankle joint during gait due to the ruptured Achilles tendon. Improving function at the ankle joint through surgery and rehabilitation increases the opportunity for the ankle joint to generate plantar flexion power in the terminal stance phase. A stable foot, with functioning tendons and muscles, may in turn also enable larger forces to be created around the knee joint. This can further enable increments in gait speed and stride length, which were found in this study. All the variables depend on each other and the increases in gait speed may also in the other direction influence the moment and power during gait. Reduced step width indicates balance improvements, since taking wider steps during gait in order to increase the support area is a common compensation strategy for impaired balance.

Compared with the healthy control group, significant impairments still persisted in patients with CATR in terms of spatiotemporal variables at the one-year follow-up. The largest impairments were slower gait speed and shorter stride length, with a mean difference of 0.24 m/s (CI 0.17–0.30) for speed and 0.16 m (CI 0.10–0.22) for stride length. However, the mean difference of 2.15% (CI − 2.94 to − 1.36) for the stance phase and 0.02 m (CI − 0.03 to − 0.01) for step width is not regarded as clinically relevant. Despite postoperative improvements in ankle moment and power, considerable deficits in power development at both the knee and ankle joints existed in patients with CATR, compared with healthy controls. Moreover, the degree of peak knee flexion was less compared with healthy controls, with a mean difference of 17.03 degrees (CI 14.75–19.31), which can be regarded as a clinically relevant difference. One reason for this could be that the preoperative compensatory neuromuscular patterns still persist, despite surgical intervention and one year of rehabilitation in patients with CATR. Patients with a CATR may need more than one year to reach their full potential for improvement and some patients may never fully recover their gait biomechanics. Functional deficits have previously been reported as persisting in patients with a CATR, ATR and Achilles tendon re-rupture (ATRR) several years after treatment, which indicates that a full recovery cannot be expected [13, 18, 26].

Previous studies have reported differences in gait biomechanics between the injured and non-injured side after treatment of ATR [15, 16]. However, impairments in gait biomechanics have also been found at the non-injured side in patients with ATR when compared with a healthy control group [15]. Thus, a comparison of the injured side with those of healthy controls was regarded as a more clinically relevant method for investigating improvements in gait biomechanics in the present study.

The primary limitation of this study is the small sample size in the CATR group. Solid conclusions cannot be drawn regarding all pre- and postoperative comparisons due to low statistical post hoc power for some variables. Moreover, mean weight and BMI differed significantly between the groups in this study, which has to be considered when interpreting the outcomes. Patients with a CATR and a higher mean body weight compared with the healthy controls, while mean height is similar between the groups, might have affected the outcomes in different ways. Soft-tissue artifacts, such as location of markers, activity performed, segment used and individual factors, are known limitation factors that can affect gait analysis results [27]. McGinley et al. [28] performed a meta-analysis of the reliability of gait analysis using an OTS, and their results showed that most errors are probably acceptable but should not be ignored.

The present study shows that surgical intervention and postoperative rehabilitation may improve gait biomechanics in patients with a CATR in terms of several important gait variables and can be an effective treatment for alterations in gait. The gait biomechanics do, however, not normalize compared with healthy controls. A total normalization of gait may therefore not be expected at one year postoperatively and whether or not gait biomechanics in patients with CATR will continue to improve after one year is not answered in this study. The long-term outcome of gait biomechanics in patients with a CATR is yet to be determined.

## Conclusion

Surgical intervention and postoperative rehabilitation for patients with a CATR resulted in improved gait biomechanics at the one-year follow-up. There were significant improvements in respect of increased waking speed and stride length, decreased step width and increased peak power in the knee and ankle joints. Gait speed, step width, stride length, stance phase and knee and ankle power still differed significantly between the patients with a CATR and healthy controls at the follow-up.

## Data Availability

The datasets used and/or analyzed in the current study are available from the corresponding author on reasonable request.

## Abbreviations

ATR: Achilles tendon rupture
ATRR: Achilles tendon re-rupture
BMI: Body mass index
CATR: Chronic Achilles tendon rupture
CI: Confidence interval
kg: Kilogram
M: Meter
Nm: Newton meter
M/s: Meters per second
ROM: Range of motion
SD: Standard deviation
W: Watt
